# Genotypic and Phenotypic Characterization of Antimicrobial Resistance Profiles in Non-typhoidal *Salmonella enterica* Strains Isolated From Cambodian Informal Markets

**DOI:** 10.3389/fmicb.2021.711472

**Published:** 2021-09-16

**Authors:** Carla L. Schwan, Sara Lomonaco, Leonardo M. Bastos, Peter W. Cook, Joshua Maher, Valentina Trinetta, Manreet Bhullar, Randall K. Phebus, Sara Gragg, Justin Kastner, Jessie L. Vipham

**Affiliations:** ^1^Department of Animal Sciences and Industry, Food Science Institute, Kansas State University, Manhattan, KS, United States; ^2^Center for Food Safety and Applied Nutrition, U.S. Food and Drug Administration, College Park, MD, United States; ^3^Department of Agronomy, Kansas State University, Manhattan, KS, United States; ^4^Centers for Disease Control and Prevention, Atlanta, GA, United States; ^5^Department of Horticulture and Natural Resources, Kansas State University, Olathe, KS, United States; ^6^Department of Diagnostic Medicine/Pathobiology, Kansas State University, Manhattan, KS, United States

**Keywords:** antimicrobial resistance, Cambodia, informal market, *Salmonella enterica*, genotypic and phenotypic characterization

## Abstract

Non-typhoidal *Salmonella enterica* is a pathogen of global importance, particularly in low and middle-income countries (LMICs). The presence of antimicrobial resistant (AMR) strains in market environments poses a serious health threat to consumers. In this study we identified and characterized the genotypic and phenotypic AMR profiles of 81 environmental *S. enterica* strains isolated from samples from informal markets in Cambodia in 2018–2019. AMR genotypes were retrieved from the NCBI Pathogen Detection website (https://www.ncbi.nlm.nih.gov/pathogens/) and using ResFinder (https://cge.cbs.dtu.dk/services/) *Salmonella* pathogenicity islands (SPIs) were identified with SPIFinder (https://cge.cbs.dtu.dk/services/). Susceptibility testing was performed by broth microdilution according to the Clinical and Laboratory Standards Institute (CLSI) standard guidelines M100-S22 using the National Antimicrobial Resistance Monitoring System (NARMS) Sensititre Gram Negative plate. A total of 17 unique AMR genes were detected in 53% (43/81) of the isolates, including those encoding tetracycline, beta-lactam, sulfonamide, quinolone, aminoglycoside, phenicol, and trimethoprim resistance. A total of 10 SPIs (SPI-1, 3–5, 8, 9, 12–14, and centisome 63 [C63PI]) were detected in 59 isolates. C63PI, an iron transport system in SPI-1, was observed in 56% of the isolates (*n* = 46). SPI-1, SPI-4, and SPI-9 were present in 13, 2, and 5% of the isolates, respectively. The most common phenotypic resistances were observed to tetracycline (47%; *n* = 38), ampicillin (37%; *n* = 30), streptomycin (20%; *n* = 16), chloramphenicol (17%; *n* = 14), and trimethoprim-sulfamethoxazole (16%; *n* = 13). This study contributes to understanding the AMR genes present in *S. enterica* isolates from informal markets in Cambodia, as well as support domestic epidemiological investigations of multidrug resistance (MDR) profiles.

## Introduction

Informal markets are common in Southeast Asian countries. In Cambodia, these markets play an important role in the country’s economy, culture, and lifestyle. In fact, a variety of food products (e.g., fresh vegetables, fruits, seafood, and animal products) are sold through these markets, which often lack basic food safety infrastructure or oversight (Roesel and Grace, 2014). Studies have shown a high prevalence of foodborne pathogens, specifically non-typhoidal *S. enterica* in food products and surfaces in the informal market settings in Cambodia (Lay et al., 2011; Trongjit et al., 2017; Nadimpalli et al., 2019).

*S. enterica* is among the top five causative agents of diarrheal diseases worldwide (World Health Organization, 2015). This pathogen has been linked to many foodborne outbreaks in several countries (Ford et al., 2018; Finger et al., 2019; Jiang et al., 2020). In the United States, approximately 94% of *S. enterica* infections are attributed to food (Hoffmann et al., 2012). Moreover, *S. enterica* is considered a pathogen of global importance, particularly in low-income countries (World Health Organization, 2017). However, surveillance data on foodborne disease is limited in Cambodia. Recently, the Mekong Institute (an intergovernmental organization founded by Southeast Asian countries) revealed that thousands of people suffer from unsafe food in Cambodia (Mekong Institute, 2019). Between 2014 and 2019, Cambodia’s Food Safety Bureau and the Department of Drug and Food (DDF) reported 134 foodborne outbreaks, resulting in 5,825 illnesses, 5,598 hospitalizations, and 81 deaths (Mekong Institute, 2019). Although informative, these estimates are likely underreported as indicated by the high percentage of illnesses that resulted in hospitalizations (96%), with the actual number of cases likely being much greater.

The lack of regulatory oversight, infrastructure, potable water, and adequate hygiene and sanitation practices have been identified as the leading causes for outbreaks (Mekong Institute, 2019) and have been previously described as a common sight among informal markets in Cambodia (Roesel and Grace, 2014). These factors often promote cross-contamination among food products, food contact surfaces and food handlers, favoring the spread of pathogens. The prevalence of *S. enterica* in the Cambodian informal market system has been previously determined, further indicating that cross-contamination is likely occurring (Schwan et al., 2020a). The ubiquity of *S. enterica* and its ability to cause human infections demonstrates that research needs to be conducted to better understand persistence and genotypic characteristics (e.g., virulence factors, antimicrobial resistance) in the serotypes present in these markets.

The indiscriminate use of antibiotics in food production (i.e., food animals) has raised immense concern among public health authorities as it contributes to the development and spread of antimicrobial resistance (AMR) among foodborne pathogens (Marshall and Levy, 2011). The spread of AMR bacteria threatens the ability to effectively treat bacterial infections, potentially leading to an extended illness period, disability, or death (World Health Organization, 2018). The World Health Organization (WHO) identified surveillance and monitoring of AMR bacteria as a global health priority (World Health Organization, 2018). Several countries (e.g., United States, Switzerland, and Australia) have conducted extensive research in identifying AMR foodborne pathogens (Barlow et al., 2015; Jans et al., 2018; Yang et al., 2020). However, limited studies have been conducted to assess the prevalence and genetic markers of AMR foodborne pathogens in Cambodia, where awareness and understanding of AMR is insufficient and needs to be investigated (Yam et al., 2019).

With the aim of understanding AMR among foodborne pathogens, the National Center for Biotechnology Information (NCBI) pathogen detection division created the NCBI Pathogen Detection database using the AMRFinder tool. This tool compares isolate genomes to a reference database of acquired resistance genes and proteins, utilizing gene hierarchy to identify the most specific protein assignment to antimicrobial resistant protein or family (Feldgarden et al., 2019). It utilizes several methods to identify AMR protein sequences or genomic nucleotide sequences. Briefly, each protein or genomic nucleotide can be identified following 100% identity and length match to a known resistance gene or allele; high identity match to a known AMR protein; partial length AMR protein sequence, or identification only through Hidden Markov Models (Feldgarden et al., 2019). These tools facilitate the identification and characterization of AMR genes from different genomes.

This study aims to identify and characterize the phenotypic and genotypic AMR profiles of *S. enterica* strains isolated from environmental samples collected at two informal markets in Cambodia between 2018 and 2019.

## Materials and Methods

### Bacterial Isolates and Sample Collection

A total of 81 *S. enterica* strains were used in this study. The strains were isolated in 2018 (June) and 2019 (January) from environmental samples collected from two informal markets in the province of Battambang, Cambodia (Schwan et al., 2020a). The strains were stored until further analysis at −80° ± 2.0°C in cryobeads (Key Scientific Products Inc., Stamford, TX-United States), following manufacturer protocol. Each isolate was assigned a unique U.S. Food and Drug Administration (FDA) Center for Food Safety and Applied Nutrition (CFSAN) identification number as part of the GenomeTrakr network (Timme et al., 2018).

### DNA Preparation

Genomic DNA from each strain was obtained using the DNeasy blood and tissue kit (Qiagen, Hilden, Germany), following manufacturer instructions. DNA concentration was determined using a Qubit 4.0 fluorometer (Invitrogen). DNA samples were sent to the FDA-CFSAN for Whole Genome Sequencing (WGS). The resultant DNA extract was stored at −20°C until WGS analysis.

### Whole Genome Sequencing and Serovar Prediction

Libraries were prepared from genomic DNA with the Nextera XT DNA Library Preparation Kit, and WGS was carried out on either the MiSeq or NextSeq sequencer, using a MiSeq Reagent Kit V2 (500-cycles) or a NextSeq 500/550 High-Output Kit V2 (300-cycles), respectively (Illumina), as previously described by Schwan et al. (2020b). *De novo* assemblies were obtained with Shovill 0.9.^1^ The serotype of each isolate was determined *in silico* using SeqSero 1.0 on draft genomes.^2^

WGS data for the 81 *S. enterica* isolates is available at NCBI under BioProject accession number PRJNA628951, as previously described by Schwan et al. (2020b).

### Detection of Antimicrobial Resistance Genes

To ensure a comprehensive analysis of known resistance genes, two database platforms were used: the NCBI Pathogen Detection (NCBI PD) and the Center for Genomic Epidemiology (CGE^3^). Under NCBI PD, AMR genes were identified using AMRFinderPlus using the following two criteria (i) Allele [100% sequence match to 100% of length to a protein named at the allele level in the Pathogen Detection Reference Gene Catalog (PDRGC)], and (ii) Exact (a 100% sequence match to 100% of length to a protein in the PDRGC that is not a named allele) (Feldgarden et al., 2019). Under CGE, AMR genes were identified using ResFinder 4.0 with the following criteria (i) ≥ 90% amino acid identity and (ii) ≥ 80% sequence length identity to known resistance proteins (Bortolaia et al., 2020). The reference databases used included acquired genes and mutations known to confer resistance to the antimicrobials aminoglycosides, β-lactams, colistin, fluoroquinolones, fosfomycin, macrolides, phenicols, rifampicin, sulfonamides, tetracyclines, and trimethoprim (Zankari et al., 2012). Salmonella pathogenicity islands (SPIs) were identified with SPIFinder (Center for Genomic Epidemiology; see text footnote 3).

### Antimicrobial Susceptibility Testing

Antimicrobial susceptibility profiles were determined by broth microdilution according to the Clinical and Laboratory Standards Institute (CLSI) standard guidelines M100-S22 (Cockerill and Clinical and Laboratory Standards Institute, 2012) using the Sensititre automated antimicrobial susceptibility system (Thermo Fisher Scientific, Waltham, MA) with the National Antimicrobial Resistance Monitoring System (NARMS) gram-negative CMV3AGNF plate (Maradiaga et al., 2017). Fourteen antimicrobials were tested: amoxicillin/clavulanic acid 2:1 ratio (AMC), ampicillin (AMP), azithromycin (AZM), cefoxitin (FOX), ceftiofur (XNL), ceftriaxone (CRO), ciprofloxacin (CIP), chloramphenicol (CHL), gentamycin (GEN), nalidixic acid (NAL), streptomycin (STR), sulfisoxazole (FIS), tetracycline (TET), and trimethoprim/sulfamethoxazole (SXT), representing nine antimicrobial classes defined by the Clinical and Laboratory Standards Institute (CLSI; Clinical and Laboratory Standards Institute, 2020). The minimum inhibitory concentration (MIC) for each antimicrobial was interpreted using the CLSI standards and NARMS breakpoints to categorize MIC results as susceptible or resistant (Food and Drug Administration, 2015; Clinical and Laboratory Standards Institute, 2020).

### Agreement Between Genotypic and Phenotypic Susceptibility

A total of 1,620 genotypic and 1,134 phenotypic data points were generated from the 81 isolates. An isolate was classified as genotypically resistant when presenting at least one gene known to confer resistance to a given antimicrobial agent, and susceptible otherwise. An isolate was classified as phenotypically resistant to a given antimicrobial agent when presenting a MIC equal to or greater than the resistant threshold based on CLSI standards and NARMS breakpoints, and susceptible otherwise. Intermediate phenotypes were considered as susceptible in this analysis. Overall and antibiotic-specific sensitivity, specificity, positive predictive value (PPV), and negative predictive value (NPV) were calculated considering genotypic (predicted) and phenotypic (observed) resistant/susceptible classification using the function *confusionMatrix* from the package caret (Kuhn, 2020) in R (R Core Team, 2020).

## Results

### AMR Resistance Genes

AMR genes were identified in 43 out of 81 isolates (53%), including those encoding tetracycline, beta-lactam, sulfonamide, quinolone, aminoglycoside, phenicol, and trimethoprim resistance. A total of 17 unique resistance genes were detected, most commonly *tet*(A) (37%, *n* = 30); *bla*_TEM__–__1_ (35%, *n* = 28); *sul2* (30%, *n* = 24); *qnrS1* (27%, *n* = 22); *aph(6)-Id* and *aph(3″)-Ib* (21%, *n* = 17 each), followed by *sul3*, and *aadA1* (17%, *n* = 14 each); *floR*, *dfrA12*, and *aadA2* (16%, *n* = 13 each); *cmlA1* (14%, *n* = 11), and *tet*(B) (10%, *n* = 8). Fewer strains presented *aph(3′)-Ia* (7%, *n* = 6); *aac(3)-IId* and *bla*_CTX__–__M__–__14_ (2%, *n* = 2 each); and *bla*_LAP__–__2_ (1%, *n* = 1) resistance genes. Among all isolates, 38 presented no AMR genes (47%).

### *Salmonella* Pathogenicity Islands

A total of 10 *Salmonella* pathogenicity islands [SPI-1, 3–5, 8, 9, 12–14, and centisome 63 (C63PI)] (Zhou et al., 1999) were detected in 59 isolates (Table 1). C63PI, an iron transport system in SPI-1, was observed in 57% of the isolates (*n* = 46). SPI-1, SPI-4 and SPI-9, which encode predicted type I and type III secretion systems (Amavisit et al., 2003; Wisner et al., 2012) were present in 16% (*n* = 13), 2% (*n* = 2), and 5% (*n* = 4) of the isolates, respectively. Twenty-seven percent of the isolates (*n* = 22) had no PIs. SPI-1 was found in *S.* Krefeld (*n* = 5), *S.* Typhimurium and *S.* Derby (*n* = 2), *S.* Hvittingfoss, *S*. Corvallis, *S.* Altona, and *S*. I 4,[5],12:i:- (*n* = 1 each). SPI-8, known to be involved with resistance to bacteriocins (Amavisit et al., 2003) was identified in *S.* Krefeld (*n* = 4), *S.* Rissen and *S.* Corvallis (*n* = 3 each).

**TABLE 1:** Serotypes, AMR genes and *Salmonella* pathogenicity islands identified in non-typhoidal *Salmonella enterica* isolates collected from Cambodian informal markets^a^.

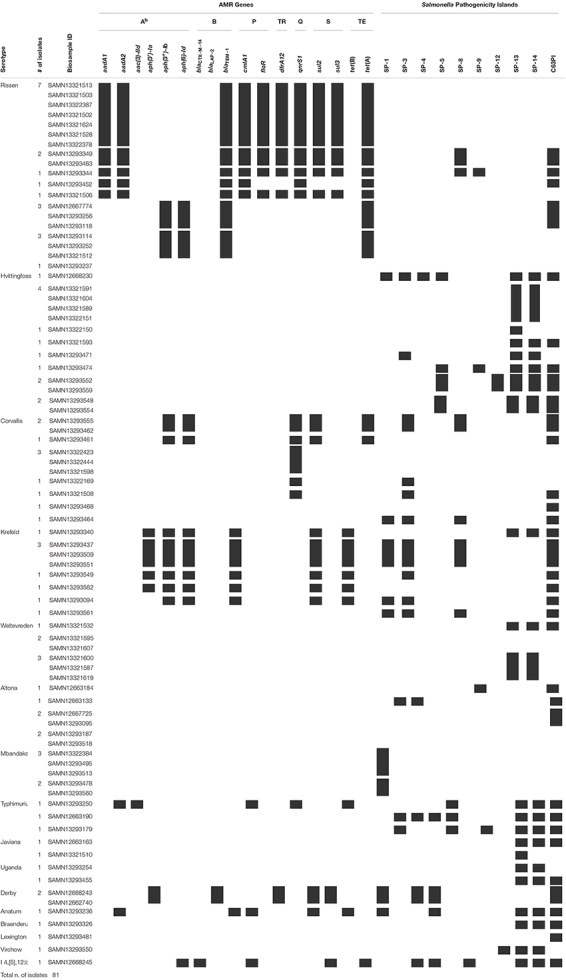

*^*a*^Black squares denote the presence of a given characteristic. ^*b*^Abbreviations of antibiotic classes: A, Aminoglycoside; B, Beta-lactam; P, Phenicol; TR, Trimethoprim; Q, Quinolone; S, Sulfonamide; TE, Tetracycline.*

### Antimicrobial Susceptibility

**FIGURE 1:**
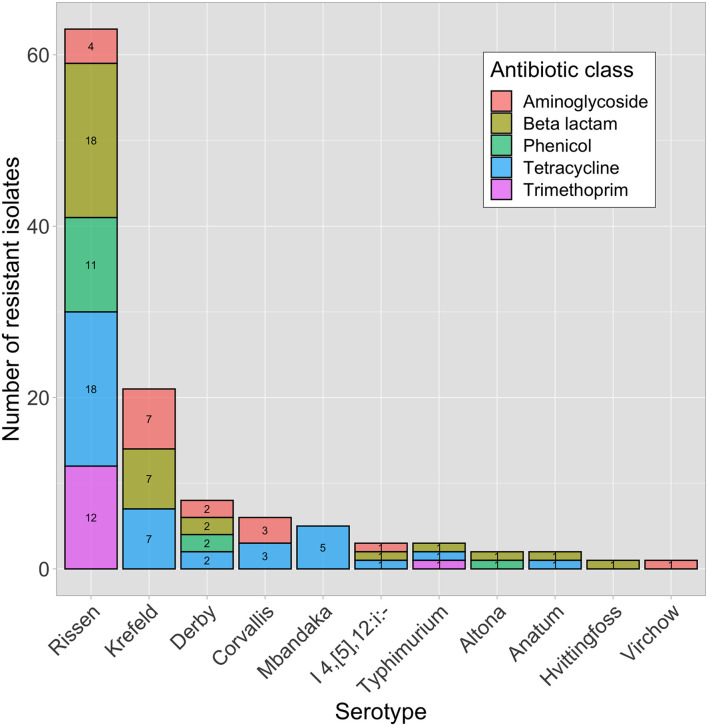
Phenotypically resistant *Salmonella enterica* isolates (*n* = 41) within different serotypes and antibiotic classes.

In total, 1,134 antibiotic tests were performed (i.e., 14 tested antimicrobial compounds for each one of the 81 isolates; Table 2) and resistance to different antimicrobials was seen in 121 instances (i.e., phenotype resistant; Table 2). Among the 81 characterized isolates, the most common phenotypically observed resistances were to tetracycline and beta-lactam (47%; *n* = 38 each), aminoglycosides (22%; *n* = 18), phenicol (17%; *n* = 14), and trimethoprim (16%; *n* = 13). No resistance was observed for quinolone, macrolide and sulfonamide (Table 3). Seventeen percent of isolates (*n* = 14) displayed resistance to three classes of antibiotics, and 16% (*n* = 13) had resistance to four of the eight classes tested. Overall, 40 isolates (49%) presented no resistance to any of the tested antimicrobial compounds. The phenotypic resistance profile by serotype and antibiotic class is shown in Figure 1. *S.* Rissen had the highest resistance diversity, displaying resistance to five classes of antibiotics, followed by *S.* Derby (4 classes), *S.* Krefeld, *S*. I 4,[5],12:i:-, *S.* Typhimurium (3 classes each), *S.* Corvallis, *S.* Altona, *S.* Anatum (2 classes each), and *S.* Mbandaka, *S.* Hvittingfoss, and *S.* Virchow (1 class each).

**TABLE 2:** Summary comparison of phenotypic and genotypic test results from 81 *Salmonella enterica* isolates from Cambodian informal markets, 2018–2019^a^.

	**No. of test results**							
	**Phenotype: resistant**		**Phenotype: susceptible**					
**Antibiotic**	**Genotype: resistant**	**Genotype: susceptible**	**Genotype: resistant**	**Genotype: susceptible**	**Sensitivity (%)**	**Specificity (%)**	**PPV (%)**	**NPV (%)**
**Aminoglycosides**								
Gentamicin (GEN)	2	0		79	100	100	100	100
Streptomycin (STR)	15	1	15	50	93.8	76.9	50.0	98.0
**Beta-lactam/beta-lactam inhibitor**								
Amoxicillin-clavulanic acid (AMC)	0	0	0	81	DBZ^b^	100	DBZ	DBZ
**Cephems**								
Cefoxitin (FOX)	0	2	0	79	0	100	DBZ	97.5
Ceftriaxone (CRO)	2	1	0	78	66.7	100	100	98.7
Ceftiofur (XNL)	2	1	0	78	66.7	100	100	98.7
**Penicillin**								
Ampicillin (AMP)	30	0	0	51	100	100	100	100
**Sulfonamide**								
Sulfisoxazole (FIS)	0	0	0	81	DBZ	100	DBZ	DBZ
**Trimethoprim**								
Trimethoprim-sulfamethoxazole (SXT)	13	0	0	68	100	100	100	100
**Macrolide**								
Azithromycin (AZM)	0	0	0	81	DBZ	100	DBZ	DBZ
**Phenicol**								
Chloramphenicol (CHL)	13	1	0	67	92.9	100	100	98.5
**Quinolones**								
Ciprofloxacin (CIP)	0	0	22	59	DBZ	72.8	DBZ	DBZ
Nalidixic acid (NAL)	0	0	0	81	DBZ	100	DBZ	DBZ
**Tetracycline**								
Tetracycline (TET)	38	0	0	43	100	100	100	100
Total	115	6	37	976	95.1	96.4	75.7	99.4

*^*a*^PPV, positive predictive value; NPV, negative predictive value.*

*^*b*^Division by zero (DBZ): values were not able to be calculated when phenotypic resistance was not observed due to division by zero.*

**TABLE 3:** Frequency of phenotypic resistance among non-typhoidal *Salmonella enterica* isolates collected from Cambodian informal markets^a^.

			**Percentage of resistant isolates (n. of isolates)**													
**Serotype**	**Multidrug resistance^b^ (n. of isolates)**	**No. of isolates tested**	**Aminoglycosides**		**Beta-lactam**					**Sulf- onamide**	**Trime- thoprim**	**Macrolide**	**Phenicol**	**Quinolone**		**Tetra- cycline**
			**GEN**	**STR**	**AMC**	**FOX**	**CRO**	**XNL**	**AMP**	**FIS**	**SXT**	**AZM**	**CHL**	**CIP**	**NAL**	**TET**
Rissen	16	19	0	21 (4)	0	0	0	0	95 (18)	0	63 (12)	0	58 (11)	0	0	95 (18)
Hvittingfoss	0	13	0	0	0	8 (1)	0	0	0	0	0	0	0	0	0	0
Corvallis	0	10	0	30 (3)	0	0	0	0	0	0	0	0	0	0	0	30 (3)
Krefeld	7	8	0	87 (7)	0	0	0	0	87 (7)	0	0	0	0	0	0	87 (7)
Weltevreden	0	6	0	0	0	0	0	0	0	0	0	0	0	0	0	0
Altona	0	6	0	0	0	17 (1)	17 (1)	17 (1)	0	0	0	0	17 (1)	0	0	0
Mbandaka	0	5	0	0	0	0	0	0	0	0	0	0	0	0	0	100 (5)
Typhimurium	1	3	0	0	0	0	0	0	33 (1)	0	33 (1)	0	0	0	0	33 (1)
Javiana	0	2	0	0	0	0	0	0	0	0	0	0	0	0	0	0
Uganda	0	2	0	0	0	0	0	0	0	0	0	0	0	0	0	0
Derby	2	2	2 (100)	0	0	0	100 (2)	100 (2)	100 (2)	0	0	0	100 (2)	0	0	100 (2)
Anatum	0	1	0	0	0	0	0	0	100 (1)	0	0	0	0	0	0	100 (1)
Braenderup	0	1	0	0	0	0	0	0	0	0	0	0	0	0	0	0
Lexington	0	1	0	0	0	0	0	0	0	0	0	0	0	0	0	0
Virchow	0	1	0	100 (1)	0	0	0	0	0	0	0	0	0	0	0	0
I 4,[5],12:i:-	1	1	0	100 (1)	0	0	0	0	100 (1)	0	0	0	0	0	0	100 (1)
Total	27	81	2 (2)	20 (16)	0	20 (2)	4 (3)	4 (3)	37 (30)	0	16 (13)	0	17 (14)	0	0	47 (38)

*^*a*^GEN, gentamicin; STR, streptomycin; AMC, amoxicillin-clavulanic acid; FOX, cefoxitin; CRO, ceftriaxone; XNL, ceftiofur; AMP, ampicillin; FIS, sulfisoxazole; SXT, trimethoprim-sulfamethoxazole; AZM, azithromycin; CHL, chloramphenicol; CIP, ciprofloxacin; NAL, nalidixic acid; TET, tetracycline.*

*^*b*^Multidrug resistant (i.e., resistance to three or more antimicrobial classes) (Food and Drug Administration, 2015).*

### Comparison Between Genotypic and Phenotypic AMR Profiles

The overall concordance between phenotypic resistance with the presence of known AMR genes was 96.2%, with genotype agreeing with phenotype for 1,091 of 1,134 of the phenotypic tests. Associated genes were predicted to cause resistance in all but six of the 121 instances in which antibiotic tests indicated resistance. These six instances of mismatch were related to beta-lactams (*n* = 4), aminoglycosides (*n* = 1), and phenicol (*n* = 1) and were observed in three serotypes (*S.* Altona, *S.* Hvittingfoss, and *S.* Virchow). This resulted in an overall sensitivity of 95% (115/121) (Table 2).

Among the 1,013 phenotypically susceptible test results, 37 belonged to isolates for which AMR genes were detected by WGS but no phenotypic resistance was observed (Table 2). Among the AMR genes detected in susceptible isolates, 15 isolates encoded aminoglycoside resistance and 22 isolates encoded quinolone resistance. This resulted in an overall specificity of 96.3% (976/1013) (Table 2). Results for each specific class of antibiotic are described in detail below and shown in Table 2.

### Resistance to β-Lactams

A total of 3 genes encoding beta-lactamases were identified, with the most common being bla_TEM__–__1_ (present in 35% of the isolates), followed by two minor genes (bla_CTX__–__M__–__14_, bla_LAP__–__2_) each present in less than 3% of isolates (Table 1). Genotypes predicted phenotypes with 89.5% sensitivity and 100% specificity.

### Resistance to Quinolone

Quinolone resistance is commonly observed within multiple mutations of the quinolone resistance-determining regions (QRDR) (i.e., gyrA, gyrB, parC, and parE) and/or one or multiple plasmid-mediated quinolone resistance (PMQR) genes (Yoshida et al., 1991; Saenz, 2003; Sorlozano et al., 2007; Hopkins et al., 2008). QRDR mutations and/or its combinations typically confer resistance to nalidixic acid and ciprofloxacin, respectively (McDermott et al., 2016; Neuert et al., 2018). Resistance mechanisms that involve a single plasmid-mediated gene typically do not confer resistance to ciprofloxacin or nalidixic acid, except when additional PMQR genes and/or QRDR mutations are present (Jacoby et al., 2014). In this study, 22 isolates carried only one PMQR gene (i.e., qnrS1) (Table 1). Resistance to ciprofloxacin or nalidixic acid was not observed in this study (thus no sensitivity was calculated for this class). Genotypic prediction for resistance resulted in a specificity of 86.4%.

### Resistance to Aminoglycosides

Six different aminoglycoside resistance alleles were identified (Table 1). Aminoglycoside phosphotransferase genes *aph(6)-Id* and *aph(3″)-Ib* (*n* = 17 each), and *aph(3′)-Ia* (*n* = 6) were identified. Genes encoding aminoglycoside adenylyltransferases were identified in 14 isolates, most commonly *aadA1* (*n* = 14) and *aadA2* (*n* = 13). Further, two isolates carried an aminoglycoside acetyltransferase *aac(3)* variant [i.e., *aac(3)-IId*] that is commonly associated with resistance to gentamicin (Tyson et al., 2017). These latter two isolates were also observed to be phenotypically resistant to the same drug. Sensitivity and specificity for genotypic-phenotypic correlations were 94.4 and 89.6%, respectively.

### Resistance to Phenicols

A combination of two genes (*floR* and *cmlA1)* from the multidrug efflux pumps family were identified in 13.6% of the isolates (*n* = 11) while two percent of the isolates only presented *floR* (*n* = 2). Genotypes predicted phenotypes with 92.9% sensitivity and 100% specificity.

### Resistance to Tetracyclines

*tet*(A) and *tet*(B) were identified herein, among the at least 40 distinct *tet* allelles described to date (Nguyen et al., 2014). Tetracycline resistance genes were found in 47% of the isolates (*n* = 38), mostly represented by *tet*(A) (*n* = 30), followed by the efflux pump-encoding *tet*(B) (*n* = 8). Genotypes predicted phenotypes with 100% sensitivity and 100% specificity.

### Resistance to Sulfonamides and Trimethoprim

Sulfonamide resistance genes were found in 33.3% of the isolates (*n* = 27). Two resistance genes (*sul2*, and *sul3*) were identified. Out of the 27 isolates, 24 carried *sul2*, and 14 carried *sul3*. Thirteen percent of the isolates (*n* = 11) had a combination of both *sul* genes. Sixteen percent of the isolates (*n* = 13) carried the dihydrofolate reductase resistance gene *dfrA12*. Genotypes predicted phenotypes with a sensitivity of 100% and a specificity of 100%.

### Resistance to Macrolides

Even though no macrolide resistance genes were identified, all isolates were phenotypically susceptible to azithromycin. Genotypic specificity for azithromycin was 100%.

### Multidrug Resistance

Among the 81 isolates analyzed, 27 (33%) were classified as multidrug resistant (MDR) as they presented resistance to three or more antimicrobial classes (Food and Drug Administration, 2015).

MDR was observed in 84% of *S*. Rissen (*n* = 16), 87% of *S.* Krefeld (*n* = 7), 100% of *S.* Derby (*n* = 2), 33% *S.* Typhimurium (*n* = 1), and 100% of *S*. I 4,[5],12:i:- (*n* = 1), shown in Table 3.

## Discussion

This study reports on the identification and characterization of the genotypic and phenotypic AMR profiles from *S. enterica* serotypes isolated from environmental samples from informal markets in Cambodia. Overall, a high diversity of resistance genes encoding resistance to several classes of antibiotics was identified.

Thirty three percent of *S. enterica* isolates (*n* = 27) presented resistance to at least three classes of antibiotics. Resistance to beta-lactams, tetracyclines, aminoglycosides, phenicol and trimethoprim were the most common among the isolates. Similarly, previous studies conducted in informal markets in Cambodia have shown that *S. enterica* isolates were resistant to β-lactams, tetracyclines, phenicol, and trimethoprim antibiotics (Trongjit et al., 2017; Nadimpalli et al., 2019). The development and spread of AMR among *S. enterica* serotypes are particularly important when found in retail environments, such as informal markets. Contaminated environments may be the source of cross-contamination between food products and environmental surfaces. When cross-contamination occurs, AMR pathogens become a threat to public health since the effectiveness of antibiotic therapy may be reduced (Thanner et al., 2016).

Twenty-seven isolates (33%) were multidrug resistant. Previous studies in Cambodia revealed that MDR *S. enterica* was found in various sample types (e.g., chicken, pork, and fish) and ranged from 23 to 52% (Trongjit et al., 2017; Nadimpalli et al., 2019). The presence of genes that are commonly associated with MDR to third generation cephalosporins (i.e., a beta-lactam class of antibiotics) is a growing problem as third generation cephalosporins are particularly important as they are often used to treat *Salmonellosis* in humans and are classified as critically important for human health (World Health Organization, 2017). In this study, 2.5% of the isolates (*n* = 2) demonstrated resistance to third generation cephalosporins (e.g., ceftriaxone and ceftiofur). Resistance to third generation cephalosporins were identified in hospitalized children (8.1%) and adults (2.7%) with *S. enterica* infections in Cambodia (Vlieghe et al., 2012; Fox-Lewis et al., 2018).

Phenotypic testing confirmed MDR in 84% of *S.* Rissen isolates and resistance to five classes of antibiotics, namely beta-lactams, tetracyclines, trimethoprim, phenicol, and aminoglycosides. Similarly, Nadimpalli et al. (2019) identified MDR *S.* Rissen from pork, chicken and fish samples collected from informal markets in Cambodia, revealing that MDR among *S.* Rissen is recurrent in the market environment. This scenario is especially concerning in low and middle-income countries (LMICs, such as Cambodia) since antibiotic resources are limited, and treatment options are compromised when strains are resistant to several classes of antibiotics.

Thirty percent of *S.* Corvallis isolates exhibited genes encoding resistance to four classes of antibiotics: aminoglycosides, quinolones, sulfonamides, and tetracyclines. However, phenotypic resistance was only observed for aminoglycosides and tetracyclines. Previous studies have reported similar AMR profiles in *S.* Corvallis isolated from various sources (i.e., informal markets, food products, and patients) from Cambodia and Thailand (Trongjit et al., 2017). The resistance pattern observed over multiple studies demonstrates that the AMR profile of *S.* Corvallis has remained similar and reoccurring over the past decade in Southeast Asian countries (e.g., Cambodia and Thailand). *S.* Corvallis has been reported as a common causative agent for travel-associated salmonellosis in patients that traveled to Southeast Asia, indicating that this serotype has recurrently caused disease (Ekdahl et al., 2005; Taguchi et al., 2009; Koch et al., 2011; Nakakubo et al., 2019).

Resistance was observed for three classes of antibiotics (i.e., beta-lactam, tetracycline, trimethoprim) in 33% of *S.* Typhimurium isolates. Similar rates of MDR have been reported from poultry and pork samples collected from informal markets and retail shops in Phnom Penh, Cambodia (Lay et al., 2011; Nadimpalli et al., 2019). In neighboring Thailand, however, higher rates (over 50%) of MDR isolates have been reported in human specimens, indicating that this strain is highly prevalent in patients (Sirichote et al., 2010).

*S.* Derby exhibited MDR in 100% of the isolates, which harbored six different genes representing six classes of antibiotics. However, resistance was observed only to four antibiotic classes: aminoglycosides, beta-lactam, phenicol, and tetracycline. These results are similar to previous studies from Cambodia (Nadimpalli et al., 2019), China (Yang et al., 2019), and Thailand (Sirichote et al., 2010; Trongjit et al., 2017), revealing that MDR is commonly found among *S.* Derby strains in Southeast Asia. Historically, *S.* Derby is mainly recovered from pork, potentially indicating that the uncontrolled use of antibiotics in the pig production chain plays an important role in the antimicrobial resistance selection pressure (Thanner et al., 2016).

The monophasic variant of *S.* Typhimurium, I 4,[5],12:i:-, has increasingly been associated with cases of human disease in several countries around the world (Moreno Switt et al., 2009). Emerging MDR clades have raised concerns among public health authorities (Elnekave et al., 2018). Sources of contamination were attributed to beef, pork, and chicken products, suggesting that these animal-based food products may serve as critical vectors for human contamination (Moreno Switt et al., 2009; Soyer et al., 2009). In this study, *S.* I 4,[5],12: i:- was resistant to aminoglycosides, beta-lactams, and tetracycline. Multidrug resistance among *S.* I 4,[5],12:i:- has also been reported from pork samples in Cambodia (Nadimpalli et al., 2019), Australia (Arnott et al., 2018), Spain (Echeita et al., 1999), and Germany (Hauser et al., 2010).

Genotypes predicted phenotypes with 100% sensitivity and 100% specificity for resistance to tetracyclines, sulfonamides and trimethoprim, and macrolides. Sensitivity of genotypic prediction was less than 100% for resistance to phenicols (92.9%), β-lactams (89.5%), quinolone (phenotypic resistance not observed), and aminoglycosides (94.4%). Specifically, in 37 isolates AMR genes were detected by WGS but no phenotypic resistance was observed. Among these resistance genes, 15 encoded aminoglycoside resistance (single variant or a combination of *aadA1*, *aadA2*, *aph(6)-Id*, *aph(3****″****)-Ib*), and 22 encoded quinolone resistance (*qnrS1*). However, those isolates exhibited phenotypic susceptibility to streptomycin (i.e., seen in 14 *S.* Rissen, 1 *S.* Anatum) and ciprofloxacin (i.e., seen in 11 *S.* Rissen, 8 *S.* Corvallis, 2 *S.* Derby, 1 *S.* Anatum). Genotype and phenotype AMR mismatches have also been observed in other studies with *Salmonella* as well as in other species, such as *Escherichia coli*, *Listeria monocytogenes*, and *Vibrio parahaemolyticus* (Deekshit et al., 2012; Ortiz et al., 2014; Lou et al., 2016; Do Nascimento et al., 2017; Neuert et al., 2018). When resistance genes are plasmid-encoded and phenotypic susceptibility testing is performed, retrospectively, these plasmids may be lost during storage and sub-culture. Additionally, these genes may be considered “silent” and several factors could be affecting the non-expression of resistance (e.g., growth medium, bacterial cell density, temperature, oxidative stress, etc.) (Udekwu et al., 2009; Poole, 2012; Lou et al., 2016; Do Nascimento et al., 2017). Therefore, even if AMR genes were detected previously by WGS, retrospective phenotypic testing on a different colony may result in a susceptible result (Neuert et al., 2018).

Additionally, three isolates (e.g., *S.* Altona, *S.* Hvittingfoss, and *S.* Virchow) were genotypically predicted to be susceptible but exhibited phenotypic resistance to beta-lactams, aminoglycosides, and phenicol. All three isolates are listed on NCBI PD as having the *mdsA* and *mdsB* genes, encoding for multidrug transporter of *S. enterica*. The *mdsABC* complex has been linked to resistance versus different drugs and toxins (Thomas et al., 2017). MdsABC, or GesABC, has been described as able to export beta-lactams, chloramphenicol, and thiamphenicol (Alcock et al., 2019; Guitor et al., 2019). Overall, this scenario highlights that alternate or emerging antimicrobial resistance mechanisms may be present, and mismatches are likely occurring due to unknown mechanisms not being included in the reference gene database used for genotypic prediction. Similar results have been observed previous studies with non-typhoidal *S. enterica* (McDermott et al., 2016; Neuert et al., 2018).

A total of 10 SPIs [SPI-1, 3–5, 8, 9, 12–14, and centisome 63 (C63PI)] were detected in 73% of isolates. Pathogenicity islands are especially important due to their capability of carrying genes encoding virulence factors that contribute to the adhesion, invasion, and infection process (Marcus et al., 2000). More specifically, C63PI, an iron transport system in SPI-1, is an important region of the *Salmonella* chromosome that mediates the entry of this species into the host cell (Ginocchio et al., 1997). To the best of our knowledge, this is the first study to explore the presence and abundance of SPIs in environmental samples from informal markets in Cambodia. SPI-1 was identified in 14% of the isolates. SPI-1, located at centisome 63 encoding predicted type III secretion system (T3SS), is a fundamental complex of genetic elements necessary during the initial stages of infection (Hardt et al., 1998; Hansen-Wester and Hensel, 2001). The T3SS machinery, which is an extremely important part of pathogenesis, facilitates the invasion by its unique needle apparatus that is utilized to deliver effector proteins into the host cell cytoplasm and create proinflammatory responses (Foley and Lynne, 2008; Meena et al., 2019). Interestingly, *S.* Krefeld, *S.* Derby and *S.* I 4,[5],12:i:- (*n* = 1 each) presented a combination of both SPI-1 and MDR, indicating that such strains are extremely important to public health due to their ability to successfully establish an infection and to resist several classes of antibiotics. The combination of an important invasion system [i.e., T3SS (SPI-1)] and the resistance profile to several different antimicrobial classes, demonstrates the importance of controlling pathogen contamination in the food chain.

Overall, this study indicates that AMR *S. enterica* serotypes are prevalent in informal market environments in Cambodia. A small percentage of the isolates was resistant to antibiotic classes commonly used to treat *S. enterica* infections in humans. Our findings revealed the presence of important SPIs related to the ability of bacteria for successful adhesion, invasion, and host cell infection. The combined effect of specific SPIs with the diversity and distribution of AMR phenotypes highlights the need for improvement in food safety practices within informal markets and the need for antibiotic stewardship in agriculture and livestock production systems.

While this study elucidated the diversity of predicted AMR genes and phenotypic resistance profiles of *S. enterica* from environmental samples, future studies are needed to identify the dissemination of AMR profiles among the food production chain. Future source-attribution studies should be conducted to investigate the dissemination of resistance among agriculture, animal food systems, and the market environment.

## Data Availability Statement

The datasets presented in this study can be found in online repositories. The names of the repository/repositories and accession number(s) can be found below: https://www.ncbi.nlm.nih.gov/, PRJNA628951.

## Author Contributions

CS, PC, VT, and JV conceived and designed the study. CS, SL, LB, and PC participated to data curation. LB and CS analyzed genotypic and phenotypic data. SL, JM, MB, SG, and JV contributed to reagents, materials, and analysis tools. CS, JV, RP, JK, and SG supervised the study and provided laboratory guidance. CS and SL prepared the manuscript. All authors revised the manuscript and approved the submitted version.

## Conflict of Interest

The authors declare that the research was conducted in the absence of any commercial or financial relationships that could be construed as a potential conflict of interest.

## Publisher’s Note

All claims expressed in this article are solely those of the authors and do not necessarily represent those of their affiliated organizations, or those of the publisher, the editors and the reviewers. Any product that may be evaluated in this article, or claim that may be made by its manufacturer, is not guaranteed or endorsed by the publisher.

## Footnotes

1
https://github.com/tseemann/shovill

2
http://denglab.info/SeqSero

3
https://cge.cbs.dtu.dk/services/
